# Trends in Shared Decision-Making Studies From 2009 to 2018: A Bibliometric Analysis

**DOI:** 10.3389/fpubh.2019.00384

**Published:** 2019-12-18

**Authors:** Cuncun Lu, Xiuxia Li, Kehu Yang

**Affiliations:** ^1^Evidence-Based Medicine Center, School of Basic Medical Sciences, Lanzhou University, Lanzhou, China; ^2^Evidence-Based Social Science Center, School of Public Health, Lanzhou University, Lanzhou, China

**Keywords:** shared decision-making, VOSviewer, CiteSpace, surgery, bibliometric analysis

## Abstract

**Background:** To systematically analyze the global development trends and research focuses of shared decision-making (SDM) studies as a reference for researchers.

**Methods:** We conducted a systematic search of the Web of Science (WoS) Core Collection on April 17, 2019, to retrieve studies related to SDM published from 2009 to 2018. VOSviewer (1.6.10), CiteSpace (5.4.R1) and Excel 2016 were used to analyze key features of SDM studies, including annual output, countries/regions, organizations, journals, authors, references, research hot-spots, and frontiers.

**Results:** Up to April 17, 2019, a total of 6,629 studies on SDM were identified as published between 2009 and 2018. The United States participated in the most studies (*n* = 3,118), with the University of California-San Francisco ranking first (*n* = 183). *Patient Education and Counseling* [impact factor (IF) 2017 = 2.785] published the most studies (*n* = 257). Legare F participated in the most studies (*n* = 101), and the paper “Charles C, 1997, Soc Sci Med, V44, P681” occupied the highest co-citation (*n* = 657) position. The research hotspots and frontiers included “Informed consent,” “Surgery,” “Depression,” “Older adult,” and “Patient-centered care.”

**Conclusion:** The number of studies concerning SDM has continued to increase since 2009, with the United States leading the field. The landscape of the basis of SDM included mainly concept, practice framework and effect assessment of SDM. “Informed consent,” “Surgery,” “Depression,” “Older adult,” and “Patient-centered care” reflected the latest research focuses, and should receive more attention.

## Introduction

Evidence-based medicine revolves around the concept that all decision-making regarding health should be based on best clinical evidence available, while emphasizing clinician experience and patient preferences and values (1, 2). When there are several alternatives, the patient's choice may determine the final treatment plan (3). Shared decision-making (SDM) is a new practical model applied to the field of healthcare that refers to clinicians working together with patients to make all decisions related to their health, including prevention, diagnosis, and treatment of conditions based on available evidence (4, 5). Several studies have showed SDM's advantages; for example, a Cochrane review (6) of more than 100 trials showed that SDM led to better outcomes in the decision-making and post-decision processes, as well as the intervention effect. In addition to improving clinical outcomes, SDM can reduce medical costs (7). Therefore, close cooperation between clinicians and patients in the development of treatment options is highly beneficial (8).

Bibliometric analysis is a scientific method that combines statistical methods with information visualization technology to identify core entities, development trends and research focuses of specific subjects or research domains. A variety of software is available that can be used for bibliometric analysis, such as VOSviewer (9) and CiteSpace (10, 11). Recently, this method has been widely employed to conduct scientometric reviews (9, 10). For example, Qiu et al. (9) employed VOSviewer to review the evolutionary process of osteoporosis in post-menopausal women. This analysis showed a gradual shift in the research focus to precision medicine-orientated “basic research.” Using CiteSpace to analyze the development trends and research frontiers in the field of Alzheimer's disease, Liu et al. (10) identified biomarkers and diagnostic criteria as the main focuses of Alzheimer's disease research.

In an analysis of publication trends of the top 15 high impact medical journals from 1996 to 2011 conducted in 2014, Blanc et al. (12) showed an exponential increase in SDM-related publications. However, this study had a number of limitations. First, the study focused on a relatively small number of high impact medical journals; therefore, important papers published in general journals may have been overlooked. Second, this study focused mainly on scientific outputs, which may limit the implications for researchers. Third, although this study was published in 2014, the analysis focused only on the period from 1996 to 2011, and so did not include any more recent findings that may have been available. Therefore, we employed the VOSviewer and CiteSpace to conduct a new bibliometric study of SDM articles and reviews indexed in the Web of Science (WoS) Core Collection as a dataset to map the global trends and research focuses in the field of SDM.

## Materials and Methods

### Data Sources

We conducted a comprehensive search of the WoS database on April 17, 2019, at Lanzhou University, Lanzhou, Gansu, China. To identify the recent developments, the timespan was set from January 1, 2009 to December 31, 2018. To ensure the representativeness of included studies, the types of publications were limited to “article” and “review.” The search strategy was as follows: Topic = “shared decision making” OR “informed decision making” OR “shared medical decision making” OR “informed medical decision making.” To avoid bias, all hits were downloaded as txt-format files from WoS on April 17, 2019 for further analysis.

### Statistical Analyses

The WoS and VOSviewer (1.6.10) (9) were used to analyze key features (annual output, countries/regions, organizations, journals, impact factor (IF), authors, and references) of the SDM-related studies retrieved on April 17, 2019. VOSviewer was used to construct a network map of countries/regions, organizations, authors, and references, respectively. CiteSpace (5.4.R1) (10, 11) was used to construct dual-map overlay of journals and explore keywords with strong burst strength. Excel (Microsoft 2016, WA, USA) was used to manage data, create other charts and all data tables. The VOSviewer settings were as follows: counting method (full counting), while, thresholds (T) of items (countries/regions, organizations, authors, and references) were adopted based on special situations. The parameters of CiteSpace were as follows: link retaining factor (LRF = 2), look back years (LBY = −1), e for top N (e = 2), time span (2009–2018), years per slice (1), links (strength: cosine, scope: within slices), selection criteria (Top 50).

## Results

### Annual Output

**Figure 1:**
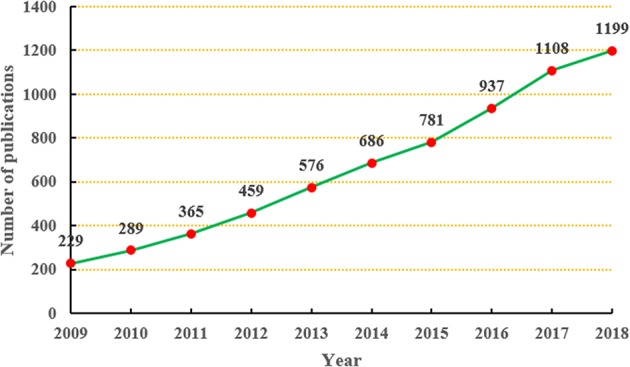
Annual output of shared decision-making studies.

In total, we retrieved 6,629 SDM-related studies published from 2009 to 2018; therefore, the average annual output was 662.9. In the 2009, only 229 studies were published; however, 10 years later in 2018, the annual output reached 1,199. As shown in Figure 1, the annual output related to SDM research showed an obvious upward trend during the period from 2009 to 2018.

### Analysis of Countries and Organizations

The top 10 countries and organizations participating in SDM studies are presented in Table 1.

**Table 1:** The top 10 countries and organizations participating in shared decision-making studies.

**Rank**	**Country/Region**	**Count**	**Organization**	**Count**
1	United States	3,118	University of California-San Francisco	183
2	England	742	Mayo Clinic	176
3	Canada	694	University of Sydney	158
4	Netherlands	574	University of Washington	143
5	Australia	516	Harvard University	136
6	Germany	506	University of Toronto	132
7	Italy	175	University of Michigan	130
8	Switzerland	171	McMaster University	120
9	France	148	University of North Carolina	119
10	Spain	144	University of California-Los Angeles	118

**Figure 2:**
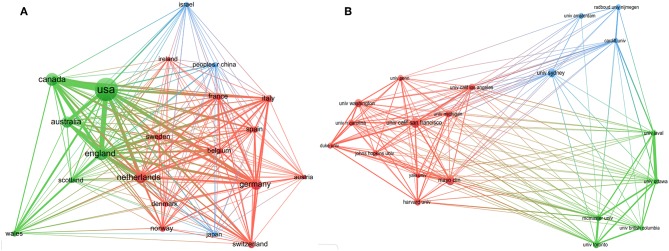
The distribution of countries (**A**, *T* = 51) and organizations (**B**, *T* = 94) participating in decision-making studies (The size of node represents the number of studies, and the thickness of line represents the degree of cooperation).

As shown in Figure 2A, each country participated in at least 144 studies related to SDM. Moreover, six (United States, England, Canada, Netherlands, Australia, and Germany) of these countries participated in at least 506 studies. Furthermore, close cooperation was observed between of these countries. Among them, the United States participated in the most studies (*n* = 3,118), followed by England (*n* = 742), Canada (*n* = 694), and Netherlands (*n* = 574).

As shown in Figure 2B, every organization participated in at least 118 studies related to SDM, with four (University of California-San Francisco, Mayo Clinic, University of Sydney and University of Washington) participated in at least 143 studies. Furthermore, close cooperation was observed between several organizations, such as the University of Ottawa and the University of Laval, McMaster University and the University of Toronto, Radboud University Nijmegen and Cardiff University, and the University of California-San Francisco and Mayo Clinic. Among them, the University of California-San Francisco ranked first, participating in 183 studies, followed by Mayo Clinic (*n* = 176) and the University of Sydney (*n* = 158).

### Analysis of Journals

**Figure 3:**
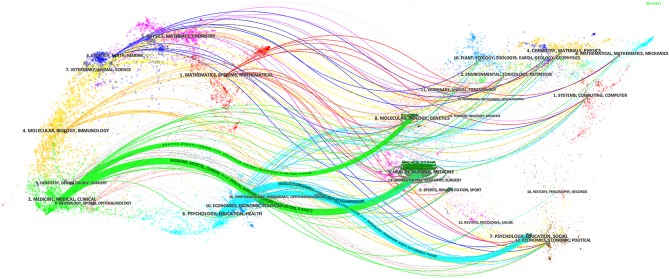
The dual-map overlay of journals publishing shared decision-making studies (Left: citing journals; Right: cited journals).

The top 10 journals publishing SDM studies are presented in Table 2. These 10 journals totally published a combined total of 915 studies related to SDM, representing ~14% of all 6,629 studies retrieved. Three journals (*Patient Education and Counseling, Health Expectations* and *PLoS ONE*) published at least 90 studies each (257, 115, and 90, respectively). In terms of IF, all 10 journals were ranked from 1.733 (*Patient Preference and Adherence*) to 4.345 (*Implementation Science*), with an average IF value of 2.721. Figure 3 shows the four citation paths. The two green paths indicate, articles published in medicine/medical/clinical journals cited journals mainly in the fields of molecular/biology/genetics and health/nursing/medicine. The other two pale blue paths indicate, articles published in psychology/education/health journals cited journals mainly in the fields of health/nursing/medicine and psychology/education/social.

**Table 2:** The top 10 journals publishing shared decision-making studies.

**Rank**	**Journal**	**Count**	**IF^†^2017**
1	Patient Education and Counseling	257	2.785
2	Health Expectations	115	2.173
3	PloS One	90	2.766
4	Medical Decision Making	83	3.012
5	BMJ Open	71	2.413
6	BMC Health Services Research	70	1.843
7	Implementation Science	63	4.345
8	BMC Medical Informatics and Decision Making	61	2.134
9	Patient Preference and Adherence	53	1.733
10	Journal of General Internal Medicine	52	4.005

† *IF, Impact Factor*.

### Analysis of Authors and References

**Figure 4:**
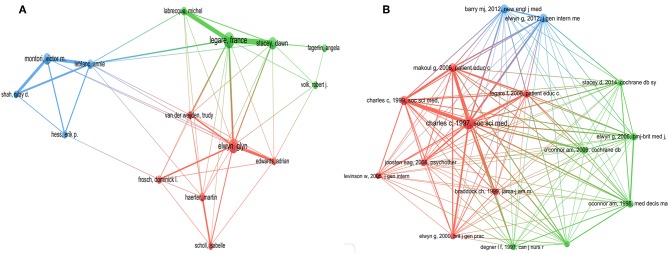
The distribution of authors (**A**, *T* = 22) and co-cited references (**B**, *T* = 152) of shared decision-making studies (The size of node represents the number of studies or co-cited number, and the thickness of line represents the degree of cooperation or co-cited strength).

The top 10 authors and co-cited references of SDM studies are presented in Table 3. As shown in Figure 4A, every author participated in at least 26 studies related to SDM; with three (Legare F, Elwyn G and Stacey D) who participated in at least 59 studies. Furthermore, close cooperation was observed between several authors, such as Legare F and Stacey D, Labrecque M and Legare F, Montori VM and Leblanc A, and Elwyn G, and Edwards A. Among them, Legare F ranked first in terms of the number of studies contributed (*n* = 101), followed by Elwyn G (*n* = 84) and Stacey D (*n* = 59). In terms of co-cited references, each reference was cited at least 192 times, with five [“Charles C, 1997, Soc Sci Med, V44, P681” (13), “Charles C, 1999, Soc Sci Med, v49, P651” (14), “Elwyn G, 2012, J Gen Intern Med, V27, P1361” (4), “Barry MJ, 2012, New Engl J Med, V366, P780” (15), “Makoul G, 2006, Patient Educ Couns, V60, P301” (16)] being cited at least 312 times. Furthermore, several references, such as “Charles C, 1997, Soc Sci Med, V44, P681” (13), “Charles C, 1999, Soc Sci Med, v49, P651” (14), and “Makoul G, 2006, Patient Educ Couns, V60, P301” (16), were cited by other articles simultaneously (Figure 4B).

**Table 3:** The top 10 authors and co-cited references of shared decision-making studies.

**Rank**	**Author**	**Count**	**Co-cited reference**	**Count**
1	Legare F	101	Charles C, 1997, Soc Sci Med, V44, P681 (13)	657
2	Elwyn G	84	Charles C, 1999, Soc Sci Med, V49, P651 (14)	334
3	Stacey D	59	Elwyn G, 2012, J Gen Intern Med, V27, P1361 (4)	319
4	Montori VM	53	Barry MJ, 2012, New Engl J Med, V366, P780 (15)	312
5	Leblanc A	33	Makoul G, 2006, Patient Educ Couns, V60, P301 (16)	312
6	Haerter M	31	Legare F, 2008, Patient Educ Couns, V73, P526 (17)	244
7	Labrecque M	28	O'Connor AM, 1995, Med Decis Making, V15, P25 (18)	239
8	Hess EP	27	Elwyn G, 2006, BMJ, V333, P417 (19)	236
9	Frosch DL	26	Joosten EA, 2008, Psychother Psychosom, V77, P219 (20)	200
10	Van Der Weijden T	26	Braddock CH, 1999, JAMA, V282, P2313 (21)	192

### Analysis of Keywords

**Figure 5:**
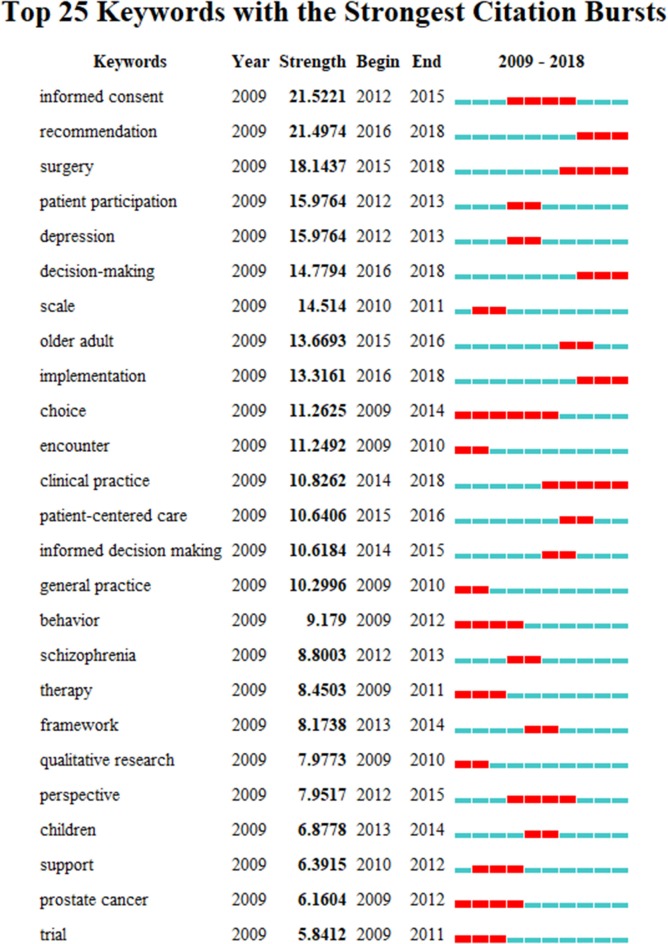
The citation burst of keywords in shared decision-making studies.

The top 20 keywords with strong burst strength in SDM studies are presented in Table 4. The keywords with strong burst strength could represent research hotspots and frontiers (11). Using CiteSpace to explore keywords with strong burst strength, we found that these keywords covered many aspects of SDM, including “older adult” and “children;” “depression” and “schizophrenia;” “informed consent,” “surgery,” “patient participation” and “patient-centered care,” and “scale,” “framework” and “qualitative research.” In terms of burst strength, “Informed consent,” “Recommendation,” “Surgery,” “Patient participation” and “Depression” had higher (*N* > 15) burst strength. In terms of end-year, the five keywords comprised “Clinical practice” (2014–2018), “Surgery” (2015–2018), “Recommendation” (2016–2018), “Decision-making” (2016–2018), “Implementation” (2016–2018), and all were published in 2018 (Figure 5).

**Table 4:** The top 20 keywords with strong burst strength in shared decision-making studies.

**Rank**	**Keyword**	**Strength**	**Rank**	**Keyword**	**Strength**
1	Informed consent	21.5221	11	Encounter	11.2492
2	Recommendation	21.4974	12	Clinical practice	10.8262
3	Surgery	18.1437	13	Patient-centered care	10.6406
4	Patient participation	15.9764	14	Informed decision making	10.6184
5	Depression	15.9764	15	General practice	10.2996
6	Decision-making	14.7794	16	Behavior	9.179
7	Scale	14.514	17	Schizophrenia	8.8003
8	Older adult	13.6693	18	Therapy	8.4503
9	Implementation	13.3161	19	Framework	8.1738
10	Choice	11.2625	20	Qualitative research	7.9773

## Discussion

### General Information

In this study, we conducted a bibliometric analysis to identify the core entities and map global trends and research focuses of SDM studies to provide a reference for researchers in this field.

We found a rapidly increasing trend in SDM studies published between 2009 and 2018, which is consistent with the findings of Blanc et al. (12) Among the top 10 countries/regions, the United States (*n* = 3,118) participated in by far the most papers and had an absolute advantage in terms of the number of studies, followed by several other countries from Western countries, such as England (*n* = 742) and Canada (*n* = 694). Meanwhile, there was also close cooperation between these countries. The possible reasons for this result are, their medical concepts and technology are often in the leading position in the world, and they have a profound knowledge accumulation. Among the top 10 organizations, the University of California-San Francisco (*n* = 183) and Mayo Clinic (*n* = 176) participated in the most papers, ranking first and second, respectively. Furthermore, among the top 10 organizations, two (University of Toronto, McMaster University) were from Canada, one (University of Sydney) was from Australia, and all the rest were located in the United States. There was also a high degree of cooperation between the organizations, such as McMaster University and the University of Toronto, and the University of California-San Francisco and Mayo Clinic. In relation to the top 10 journals, *Patient Education and Counseling* (*n* = 257, IF2017 = 2.785) and *Health Expectations (n* = *115*, IF2017 = 2.173) published the most papers, ranking first and second. *Implementation Science* (*n* = 63, IF2017 = 4.345) had the highest IF, while the IFs of *Patient Education and Counseling* and *Health Expectations* ranked in only a medium position. Moreover, most of the journals focused on public health or medicine, and usually cited papers from the fields of health or social science. Among the top 10 authors, Legare F (*n* = 101), Elwyn G (*n* = 84), and Stacey D (*n* = 59) ranked first, second, and third, respectively, and we found close cooperation between Legare F and Stacey D. Moreover, Legare F and Elwyn G were authors of the most co-cited documents (4, 17, 19). These results can provide guidance for beginners in this field to cooperate with other entities, and submit their manuscripts.

### Intellectual Base

A document co-citation network represents how frequently two studies are cited together by other papers (10, 11). These co-cited studies could be regarded as intellectual base of a special field or subject, which is also known as knowledge base (10, 11). Thus, we focused on the top 10 co-cited studies to evaluate the intellectual base related to SDM.

The most cited study with the highest number of co-citations (*n* = 657) was published in 1997 by Charles et al. (13). This study provided a clearer definition of SDM by identifying the following four key features: (1) two objects (clinician and patient) must be involved; (2) they should share information that they have; (3) they should reach a consensus based on treatment preference; and (4) reaching consensus on the treatment to practice. The second most cited paper received 334 co-citations, and also was published by Charles et al. (14), this paper revisited their framework on SDM, and raised decision-making approaches between three predominant models of SDM. The third most cited paper was published in 2012 by Elwyn et al. (4), with 319 co-citations. This paper proposed a specific three-step model of practice SDM, involving introduction of choice, description of alternatives, and helping patients to explore their preference and aid in their decision-making. The fourth most cited study, with 312 co-citations, was published in 2012 by Barry et al. (15). These authors proposed that SDM as the central tenet of patient-centered care was long overdue, and recommended that clinicians should view the healthcare experience through their patient's eyes. Makoul and Clayman (16) published the fifth most cited study in 2006, with 312 co-citations. This systematic review determined the range of SDM definitions, and eventually, an interactively conceptual definition was outlined. Legare et al. published the sixth most cited paper (17) in 2008, with 244 co-citations. This article reported an updated systematic review aiming to identify the barriers and facilitators associated with the implementation of SDM in clinical practice based on the opinions of health professionals regarding knowledge, attitudes and behaviors, such as inadequate awareness applicability, motivation, and communication. O'Connor (18) published the seventh most commonly co-cited paper, which reported the acceptability if using psychometric properties of the decisional conflict scale for application in SDM. Elwyn et al. (19) published the eighth most commonly co-cited paper, which reported the development of quality criteria for decision aids using the Delphi method. The authors established a quality criterion framework containing 12 unique domains. Joosten et al. (20) published the ninth most commonly co-cited paper, describing a systematic review of the effects of SDM on patient outcomes. This group found that SDM had no significant impact on patient outcomes in the short-term and suggested that future studies should focus on long-term decisions. Braddock et al. (21) published the tenth most commonly co-cited paper, reporting an evaluation of the nature and completeness of informed decision making in a cross-sectional study including 1,057 patients and 59 clinicians. These authors reported inadequacies in the efforts to encourage informed decision-making in clinical practice. Overall, we found that the top 10 co-citations focused mainly on aspects, such as concept or definition, practice framework or steps, and effect assessment of SDM, all which are the foundations of SDM research.

### Research Hotspots and Frontiers

The keywords with strong burst strength are implicated as those that are paid special attention by scientific community during a specific time-period, and could therefore represent research hotspots and frontiers of a special field or subject in one period (11). Therefore, five phrases with higher burst were selected for in-depth analysis: “Informed consent,” “Surgery,” “Depression,” “Older adult,” and “Patient-centered care.”

“Informed consent” is a dynamic process used to build mutual trust, in which the clinicians communicate with the patients to ensure that the patients know his or her own treatment decisions, and it usually includes oral communication and text material (22). A cross sectional study showed that age and level of education may influence the adequacy of written informed consent for surgical patients (22). Especially, a recent article (23) highlighted that the concept of traditional “Informed Consent” is evolving to match the requirements of the relatively new era of SDM.

“Surgery,” which usually refers to manipulations implemented for the correction of abnormal conditions, repair of injuries, and treatment of particular diseases, is a complex and potentially dangerous intervention (24). It has been estimated that ~234.2 million surgical operations are performed every year worldwide (24). The efficiency of surgery is often influenced by a number of factors, such as the surgeon's level of experience, decision-making, teamwork and environment (25). Therefore, informed consent and SDM are particularly critical in surgery. Efforts have been made to improve the communication and reporting of informed consent in surgery. For example, a current project (26) is underway to develop a core outcome set to improve reporting of outcomes and consent processes. A systematic review to identify features of SDM in surgery conducted by de Mik et al. (27) showed that surgeons and patients both recognized SDM, although SDM in surgery is in its infancy. Therefore, strategies to effectively practice and assess SDM in surgery are particularly important.

“Depression” is a common and heterogeneous condition with a chronic and recurrent natural course, that usually influences psychosocial functions and lowers quality of life of suffers (28). And according to the report from World Health Organization in 2017, there are more than 300 million people, or 4.4% of the global population (28). The high prevalence of depression leads to an enormous social and economic burden; therefore, strategies to control its occurrence are particularly important. A cluster randomized trial (29) including 117 clinicians and 301 patients showed that SDM improved the process of decision-making and the quality of primary care for patients with depression. Similarly, an uncontrolled cohort study (30) showed that SDM improved the process of decision-making and reduced depression symptoms in young patients.

“Older adult” is a special patient group that is often affected by multimorbidity. This group is increasing in size with the aging population and represents a huge social burden (31). In a cross-sectional investigation (32), SDM was shown to offer an approach to orthopedic care that is highly consistent with the preferences of older patients. SDM is regarded as a fundamental component of “Patient-centered care” (33). Ironically, despite the current information explosion, most patients have insufficient information about their condition and corresponding interventions for a variety of reasons. In 2013, the Institute of Medicine (IOM) found that cancer patients and their families could not fully understand adequate information to make informed decisions about their care in the United States, and highlighted the need for better patient-clinician communication and improved care coordination for this situation (34). Elwyn et al. (35) hypothesized that different methods of patient-centered care are needed depending on various clinical situations, and proposed a combination of motivational interviewing and SDM to achieve patient-centered care based on the patient's preferences and values.

### Strengths and Limitations

The present study comprehensively analyzes the global trends and status of SDM research over the past decade by using the scientific method of bibliometric analysis. We systematically searched the WoS, and downloaded all relevant data on the same day. The core countries/regions, organizations, authors, journals, and research focuses were then identified to provide reference for scientists in the SDM field.

However, our study has some limitations like other scientometric studies. Firstly, we searched only the WoS, and other large databases such as PubMed, EmBase and Scopus were not included, which may lead to the omission of some important studies from the present dataset. However, most published key papers are included in the WoS database, and moreover, the WoS is the most commonly used database in bibliometrics analysis (9, 10). Secondly, all datasets were identified using computer software or tool, such as VOSviewer, CiteSpace, and WoS, rather than being selected and collected manually, and manual selection and collection of datasets is required for systematic reviews or overviews (36–40); therefore, these datasets may be subject to bias (e.g., irrelevant studies may be included), although this would not affect the trends identified and conclusions of the present study. Finally, data generated from studies published in the current year (2019) were not included in our analysis as the dataset for this year is incomplete.

## Conclusion

In the present bibliometric study, we identified a continual increase in the number of SDM-related studies since 2009, with the United States leading the field. *Patient Education and Counseling* was the core journal and Legare F was the most active author. The landscape of the basis of SDM included mainly concept, practice framework and effect assessment of SDM. “Informed consent,” “Surgery,” “Depression,” “Older adult,” and “Patient-centered care” reflected the latest focuses, and should receive more attention.

## Data Availability Statement

The data used to support the findings of this study can be requested from the corresponding author.

## Author Contributions

KY and CL designed this study. CL and XL ran search strategy, collected data, and performed analyses. KY re-checked. CL wrote the manuscript. KY, CL, and XL reviewed the manuscript.

### Conflict of Interest

The authors declare that the research was conducted in the absence of any commercial or financial relationships that could be construed as a potential conflict of interest.
